# Prognostic Factors in Interstitial Lung Disease Associated with Primary Sjögren’s Syndrome: A Retrospective Analysis of 33 Pathologically–Proven Cases

**DOI:** 10.1371/journal.pone.0073774

**Published:** 2013-09-09

**Authors:** Yasunori Enomoto, Tamiko Takemura, Eri Hagiwara, Tae Iwasawa, Yuh Fukuda, Noriyo Yanagawa, Fumikazu Sakai, Tomohisa Baba, Shouhei Nagaoka, Takashi Ogura

**Affiliations:** 1 Department of Respiratory Medicine, Kanagawa Cardiovascular and Respiratory Center, Yokohama, Japan; 2 Department of Pathology, Japanese Red Cross Medical Center, Tokyo, Japan; 3 Department of Radiology, Kanagawa Cardiovascular and Respiratory Center, Yokohama, Japan; 4 Department of Analytic Human Pathology, Nippon Medical School, Tokyo, Japan; 5 Department of Radiology, Tokyo Metropolitan Cancer and Infectious Diseases Center Komagome Hospital, Tokyo, Japan; 6 Department of Diagnostic Radiology, Saitama International Medical Center, Saitama Medical University, Saitama, Japan; 7 Department of Rheumatology, Yokohama Minami Kyosai Hospital, Yokohama, Japan; Kliniken der Stadt Köln gGmbH, Germany

## Abstract

**Introduction:**

Interstitial lung disease associated with primary Sjögren’s syndrome (pSS–ILD) shows several patterns such as nonspecific interstitial pneumonia (NSIP) and usual interstitial pneumonia (UIP). Although UIP is a well–recognized prognostic determinant in idiopathic interstitial pneumonias, whether this is also the case in pSS–ILD is unclear. The objectives of this study were to evaluate the prognostic effect of UIP, and to identify the prognostic factors in pSS–ILD.

**Methods:**

A retrospective review of medical records identified 33 consecutive patients with pathologically–proven pSS–ILD. Each patient was classified into each ILD pattern by multidisciplinary analysis. Baseline clinical–radiologic–pathologic characteristics and survival rates were compared between the ILD patterns. Finally, the prognostic factors in pSS–ILD were assessed by univariate and subsequent multivariate analyses using Cox’s proportional hazards regression model.

**Results:**

pSS–ILD patients were diagnosed with NSIP (n = 22) or UIP (n = 11). The median follow–up period was 110 months, and five-year survival rate was 87.3% in the total patient population. The prognosis of the UIP patients was not significantly different from that of the NSIP patients (NSIP to UIP, hazard ratio [HR]: 0.77, 95% confidence interval [CI]: 0.18–3.36, *P* = 0.73). Multivariate analysis identified PaCO_2_ (HR: 1.68 per 1 Torr increase, 95% CI: 1.24–2.28, *P* < 0.01), extent of reticular abnormality on high–resolution CT (HR: 4.17 per 1-grade increment, 95% CI: 1.18–14.73, *P* = 0.03), and severity of fibroblastic foci (HR: 9.26 per 1-grade increment, 95% CI: 1.74–49.35, *P* < 0.01) as prognostic factors in pSS–ILD.

**Conclusions:**

UIP in pSS–ILD was not related to poorer prognosis than NSIP. Assessment of detailed clinical–radiologic–pathologic findings is more important than distinguishing UIP to evaluate prognosis in this disease.

## Introduction

Primary Sjögren’s syndrome (pSS) is a chronic inflammatory autoimmune disease characterized by deteriorated salivary and lacrimal gland function and associated lymphocytic infiltration of exocrine glands. Among a variety of organs affected in pSS, lung involvement is one of the most common extraglandular complications with a prevalence of 9%–75% [1,2]. In a recent report, pSS patients with lung involvement had increased risk of death in comparison with those without lung involvement [2]. Therefore, understanding the characteristics of lung involvement is essential for management of pSS.

The classification of interstitial lung disease (ILD) associated with collagen vascular diseases including pSS is temporally based on the 2002 American Thoracic Society/European Respiratory Society consensus classification of idiopathic interstitial pneumonias (2002 ATS/ERS classification of IIPs) [3]. ILD is the most frequent lung involvement in pSS, and known to show several patterns such as nonspecific interstitial pneumonia (NSIP) and usual interstitial pneumonia (UIP) [4]. There are only a few studies focusing on pathologically–proven ILD associated with pSS (pSS–ILD) [5–7]. Although UIP is known to be a poorly prognostic determinant in IIPs [8], whether this is also the case in pSS–ILD is unclear. Moreover, the prognostic factors in pSS–ILD still remain unknown. In this retrospective study, our objectives were to examine whether UIP was associated with poor prognosis as recognized in IIPs, and to identify the prognostic factors in pathologically–proven pSS–ILD.

## Methods

### Study Subjects

This study was approved by the institutional review board of Kanagawa Cardiovascular and Respiratory Center. Because of the retrospective nature of the study, the review board waived the need for written informed consent from the patients.

We retrospectively analyzed the medical records of 396 consecutive patients who underwent surgical lung biopsy for diagnosis of diffuse lung diseases at Kanagawa Cardiovascular and Respiratory Center between November 1998 and November 2008. Of those, 38 patients fulfilled the diagnostic criteria for pSS [9] at the time of lung biopsy. Diagnosis of pSS by rheumatologists was confirmed from medical record reviews. On the next step, we excluded five patients with lung involvement other than pSS–ILD: amyloidosis, malignant lymphoma, bronchiolitis obliterans, nonspecific bronchiolitis alone, and pneumoconiosis.

### Clinical Analysis

We extracted demographic data, clinical presentation, physical findings, and laboratory findings at the time of surgical lung biopsy from medical records. We reviewed the results of pulmonary function tests performed before the date of biopsy, which were obtained from all eligible patients (n = 33). The median interval between the date of biopsy and pulmonary function tests was 22 days (range: 4–200 days). Bronchoalveolar lavage (BAL) was performed in 27 of the 33 patients, and the median interval from the date of biopsy was 21 days (range: 8–285 days).

Information regarding vital status and causes of death were obtained from medical records. Survival time was calculated from the date of lung biopsy until the end of the follow–up period.

### Radiological Analysis

Chest high-resolution computed tomography (HRCT) scans before surgical lung biopsy were obtained from all the 33 patients at full inspiration with 1.0- or 2.0-mm–thick sections throughout the entire lungs. The median interval between the date of biopsy and HRCT was 15 days (range: 1–148 days). All images were reviewed independently without knowledge of clinical and pathological information by two experienced radiologists.

The following HRCT findings were assessed for the presence or absence in whole lung scans: honeycombing, bronchiectasis, and dilatation of pulmonary artery. The extent of reticular abnormality (reticulation and honeycombing), ground glass attenuation (increased lung attenuation in the absence of reticular abnormality), consolidation, and micronodules were semi-quantitatively scored from grade 0 (0% of lung parenchyma), 1 (< 10%), 2 (10%–25%), to 3 (> 25%) at four levels of horizontal axial view in each lung. The first level was defined as at the aortic arch, the second level as at the carina tracheae, the third level as at the right pulmonary vein, and the fourth level as at the top of the right diaphragm. The selection of these factors and the manner of assessment were determined by references to past studies [10–14].

Subsequently, each scan was classified as UIP pattern, NSIP pattern, or others on the basis of the 2002 ATS/ERS classification of IIPs [3]. Disagreements between two radiologists were resolved by consensus.

### Pathological Analysis

Histological sections of surgical lung biopsy specimens from all the 33 patients were stained with hematoxylin–eosin and elastic van Gieson. Multiple specimens mainly from upper and lower lobes were available in 21 of the 33 patients. All slides were reviewed independently by two experienced lung pathologists who were not aware of the clinical and radiological findings.

The following pathological features were semi-quantitatively graded as 0 (absent), 1 (mild), 2 (moderate), to 3 (severe): interstitial inflammation, interstitial fibrosis, lymphoid follicle with germinal center, organizing pneumonia, fibroblastic foci, microscopic honeycombing, cellular bronchiolitis, bronchiolar fibrosis (fibrosis around terminal and respiratory bronchioles), and vascular intimal or medial thickening. Pathological assessment was referred to previous studies [15–17].

Subsequently, the pathological ILD pattern was determined in accordance with the 2002 ATS/ERS classification of IIPs for each patient [3]. When two or more patterns coexisted, major one and minor ones were described. Disagreements between two pathologists were discussed until consensus was reached.

### Multidisciplinary Diagnosis

Clinical, radiological, and pathological data were all gathered, and multidisciplinary diagnosis of the ILD pattern was determined for each patient with discussion by pulmonologists, radiologists, and pathologists in a general conference. Cases with differing diagnoses between radiological and pathological assessment were particularly discussed in detail and diagnosed by consensus.

### Statistical Analysis

Group comparison was conducted by Student’s t–test, Mann–Whitney’s U–test, or Fisher’s exact test, as appropriate. Survival time was calculated as the number of months from the date of surgical lung biopsy until the date of death or censoring. Patients were censored if they became lost to follow–up or were alive on July 3, 2012. The Kaplan–Meier method was used to produce the survival curve, and log–rank test was performed to compare the prognosis between the ILD patterns. Univariate and subsequent multivariate analyses using Cox’s proportional hazards regression model were performed to evaluate the prognostic effect of UIP, and to identify the prognostic factors in pSS–ILD. All *P* values < 0.05 were considered statistically significant. Statistical analyses were performed using SPSS software version 13.0 (SPSS, Inc., Chicago, IL, USA).

## Results

### Diagnosis of pSS–ILD

The summary of ILD patterns of all the 33 patients is listed in Table 1. Two or more pathological patterns such as UIP combined with fibrotic NSIP were often observed. NSIP was the most frequent in multidisciplinary diagnosis (22 of 33 patients: 67%); of those, fibrotic NSIP was more common than the cellular one. The others were all diagnosed as UIP (11 of 33 patients: 33%), which was not rare in this cohort.

**Table 1:** HRCT patterns, pathological patterns, and multidisciplinary diagnosis of the 33 patients with interstitial lung disease associated with primary Sjögren’s syndrome.

**HRCT Pattern**	**Pathological Pattern**	**Multidisciplinary Diagnosis**
	**Major > Minor**	
UIP (n = 11)	UIP (n = 3)	UIP (n = 11)
	UIP > f–NSIP (n = 8)	
f–NSIP (n = 17)	f–NSIP > UIP (n = 4)	NSIP (n = 22)
	f–NSIP > UIP, OP (n = 1)	f–NSIP (n = 19)
	f–NSIP (n = 10)	c-NSIP (n = 3)
	c and f–NSIP (n = 1)	
	f–NSIP (n = 1)	
c-NSIP (n = 3)	c and f–NSIP > Bronchiolitis (n = 1)	
	f–NSIP (n = 1)	
	c-NSIP (n = 1)	
Unclassifiable (n = 2)	f–NSIP > Bronchiolitis (n = 1)	
	c-NSIP > Bronchiolitis (n = 1)	

HRCT = high–resolution computed tomography; UIP = usual interstitial pneumonia; NSIP = nonspecific interstitial pneumonia(c– = cellular; f– = fibrotic); OP = organizing pneumonia

### Clinical Findings

Twelve patients had been diagnosed with pSS before the first visit to our institution, and the median disease period was 20.5 months (range: 3–132 months). In contrast, pSS was diagnosed in the other 21 patients at the time of ILD diagnosis, suggesting that ILD could be the first symptom of pSS.

Baseline clinical characteristics are listed in Table 2. Median age was 66 years, with females and never–smokers predominating in the entire patient population. Age, sex, and smoking history were not significantly different between the NSIP and UIP patients. Anti SS–A/Ro antibody positivity was significantly more frequent in the UIP patients (*P* = 0.02).

**Table 2:** Clinical characteristics and laboratory test results of the patients with interstitial lung disease associated with primary Sjögren’s syndrome and comparison of those variables between the NSIP and UIP patients.

**Variables**	**All Patients**	**NSIP**	**UIP**	***P***
Age, y*	66, 62–71	64, 60–71	68, 66–71	0.16
	(n = 33)	(n = 22)	(n = 11)	
Female	23	16	7	0.70
	(n = 33)	(n = 22)	(n = 11)	
Never smoker	23	17	6	0.24
	(n = 33)	(n = 22)	(n = 11)	
Dry eyes or dry mouth	29	19	10	1.00
	(n = 31)	(n = 20)	(n = 11)	
BMI, kg/m^2^	23.7 ± 3.8	24.0 ± 4.1	23.3 ± 3.1	0.66
	(n = 33)	(n = 22)	(n = 11)	
Anti SS–A/Ro antibody, positive	20	10	10	0.02
	(n = 33)	(n = 22)	(n = 11)	
Anti SS–B/La antibody, positive	9	6	3	1.00
	(n = 32)	(n = 22)	(n = 10)	
LDH, IU/L	215 ± 54	220 ± 62	205 ± 32	0.47
	(n = 32)	(n = 22)	(n = 10)	
KL-6, U/mL (reference range < 500)	1308 ± 855	1450 ± 970	1025 ± 500	0.26
	(n = 24)	(n=16)	(n = 8)	
IgG, mg/mL	2049 ± 889	2026 ± 1040	2084 ± 623	0.88
	(n = 26)	(n = 16)	(n = 10)	
PaO_2_, Torr (room air)	74.5 ± 7.2	74.3 ± 7.1	75.0 ± 7.8	0.80
	(n = 33)	(n = 22)	(n = 11)	
PaCO_2_, Torr (room air)	41.1 ± 4.7	41.5 ± 5.1	40.3 ± 3.9	0.53
	(n = 33)	(n = 22)	(n = 11)	

Data are presented as n, mean ± standard deviation, or median with interquartile range, depending on distribution. All *P* values were evaluated by comparing between the NSIP and UIP patients using Fisher’s exact test, Student’s t–test, or Mann–Whitney’s U–test, as appropriate. NSIP = nonspecific interstitial pneumonia; UIP = usual interstitial pneumonia; BMI = body mass index; LDH = lactate dehydrogenase; KL-6 = Krebs von den lungen-6; IgG = immunoglobulin G; PaO_2_ = arterial oxygen pressure; PaCO_2_ = arterial carbon dioxide pressure.

* At the time of surgical lung biopsy.

The results of pulmonary function tests and BAL are summarized in Table 3. Forced vital capacity (FVC) percentage predicted (% pred) in the NSIP patients was relatively lower than that in the UIP patients. Forced expiratory volume in one second % pred in the NSIP patients was significantly worse than that in the UIP patients (*P* = 0.02). BAL findings between the NSIP and UIP patients did not significantly differ, although proportion of lymphocytes was relatively higher in the NSIP patients.

**Table 3:** Pulmonary function tests and bronchoalveolar lavage fluid analyses of the patients with interstitial lung disease associated with primary Sjögren’s syndrome and comparison of those variables between the NSIP and UIP patients.

**Variables**	**All Patients**	**NSIP**	**UIP**	***P***
**Pulmonary function**				
FVC % pred, %	86.3 ± 25.8	80.2 ± 25.2	98.5 ± 23.5	0.05
	(n = 33)	(n = 22)	(n = 11)	
FEV_1_ % pred, %	95.3 ± 27.7	87.4 ± 25.4	111.1 ± 26.4	0.02
	(n = 33)	(n = 22)	(n = 11)	
DLco % pred, %	84.5 ± 21.5	82.8 ± 20.7	88.0 ± 23.7	0.56
	(n = 27)	(n = 18)	(n = 9)	
**Bronchoalveolar lavage fluid**				
Total cells, /μl	284 ± 226	278 ± 196	296 ± 290	0.86
	(n = 24)	(n = 16)	(n = 8)	
Lymphocytes, %	27.7 ± 23.7	33.0 ± 26.6	17.1 ± 11.8	0.10
	(n = 27)	(n = 18)	(n = 9)	
CD4/8 ratio	0.8, 0.3–1.6	0.7, 0.3–1.5	1.0, 0.5–2.2	0.39
	(n = 26)	(n = 17)	(n = 9)	

Data are presented as mean ± standard deviation or median with interquartile range depending on distribution. All *P* values were evaluated by comparing between the NSIP and UIP patients using Student’s t–test or Mann–Whitney’s U–test, as appropriate. NSIP = nonspecific interstitial pneumonia; UIP = usual interstitial pneumonia; FVC = forced vital capacity; FEV_1_ = forced expiratory volume in one second; DLco = diffusion capacity for carbon monoxide; pred = predicted.

### Radiological and Pathological Findings

**Figure 1:**
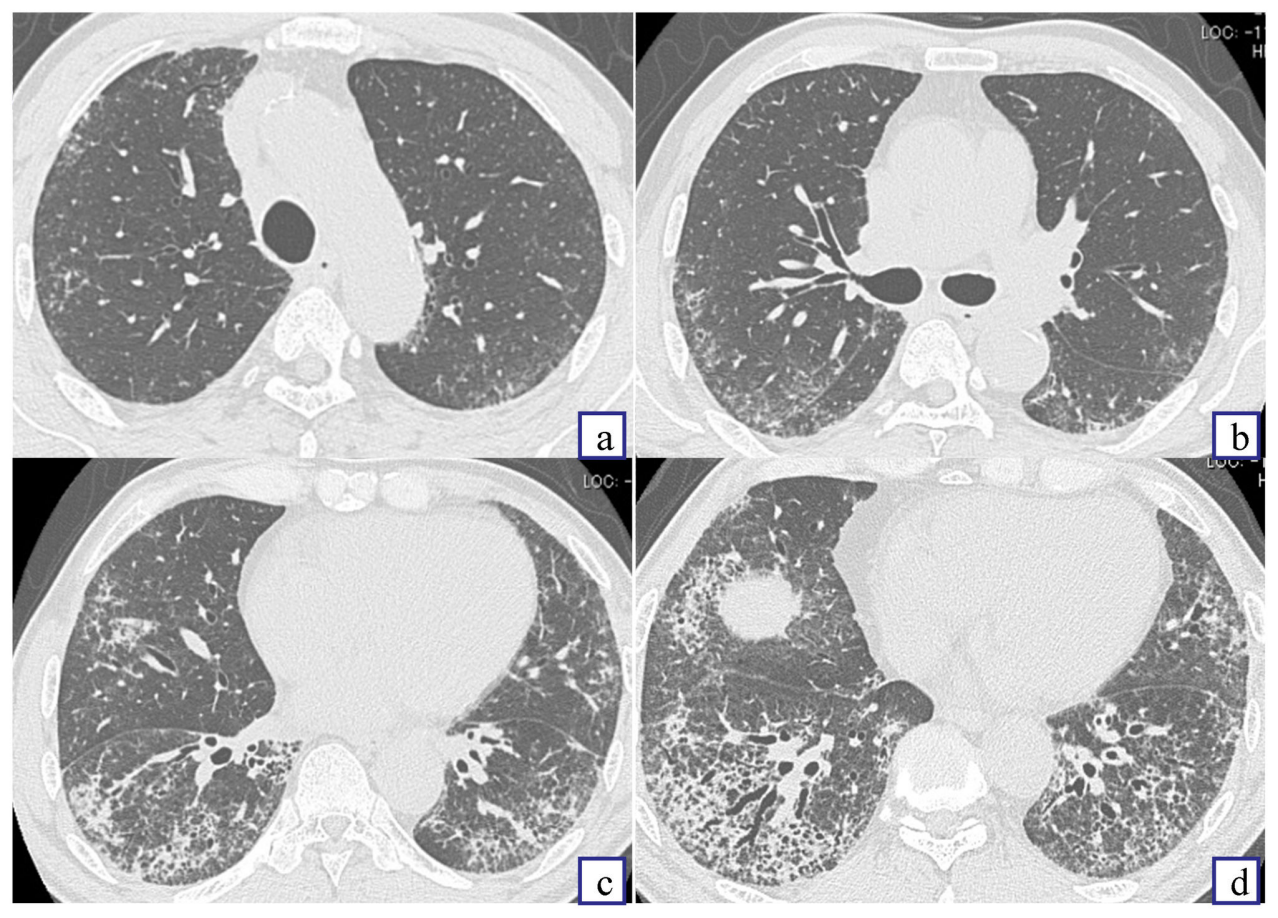
An example of high–resolution CT images. Analysis of each finding was as follows. Honeycombing: negative; Bronchiectasis: positive; Dilatation of pulmonary artery: negative; Extent of ground glass attenuation: grade 1; Extent of consolidation: grade 1; Extent of reticular abnormality: grade 2; Extent of micronodules: grade 2. This case was diagnosed as fibrotic nonspecific interstitial pneumonia pattern. (a) at the aortic arch. (b) at the carina tracheae. (c) at the right pulmonary vein. (d) at the top of the right diaphragm.

A summary of HRCT findings is shown in Table 4. Ground glass attenuation and reticular abnormality were found in almost all patients, and the distribution was frequently bilateral and lower dominant (data not shown). Honeycombing was significantly more common in the UIP patients than in the NSIP patients (*P* < 0.01). Consolidation was observed only in the NSIP patients. An example of HRCT scans and the results of analysis are shown in Figure 1.

**Table 4:** HRCT and pathological findings of the patients with interstitial lung disease associated with primary Sjögren’s syndrome and comparison of those variables between the NSIP and UIP patients.

**Variables**	**NSIP**	**UIP**	***P***
**(n = 33)**	**(n = 22)**	**(n = 11)**	
**HRCT findings**			
Honeycombing, positive	2	6	< 0.01
Bronchiectasis, positive	18	11	0.28
Dilatation of pulmonary artery, positive	8	1	0.21
Ground glass attenuation, Grade 0/1/2/3	2/14/3/3	1/8/1/1	0.65
Consolidation, Grade 0/1/2/3	14/7/1/0	11/0/0/0	0.02
Reticular abnormality, Grade 0/1/2/3	2/12/7/1	0/6/4/1	0.43
Micronodules, Grade 0/1/2/3	6/12/3/1	4/3/3/1	0.78
**Pathological findings, Grade 0/1/2/3**			
Interstitial inflammation	0/7/14/1	0/4/7/0	0.68
Interstitial fibrosis	0/6/14/2	0/3/6/2	0.73
Lymphoid follicle with germinal center	3/9/9/1	0/7/2/2	0.76
Organizing pneumonia	6/11/4/1	0/11/0/0	0.80
Fibroblastic foci	7/11/1/3	1/5/5/0	0.12
Microscopic honeycombing	15/5/1/1	2/5/3/1	< 0.01
Cellular bronchiolitis	1/12/7/2	0/6/5/0	0.88
Bronchiolar fibrosis	11/7/4/0	5/5/1/0	0.97
Vascular intimal or medial thickening	6/7/8/1	1/3/6/1	0.17

Data are presented as n. All *P* values were evaluated by comparing between the NSIP and UIP patients using Mann–Whitney’s U–test or Fisher’s exact test, as appropriate. The higher grade means the larger extent on HRCT or more severe change in pathological assessment (see Methods section for detail). HRCT = high-resolution computed tomography; NSIP = nonspecific interstitial pneumonia; UIP = usual interstitial pneumonia.

**Figure 2:**
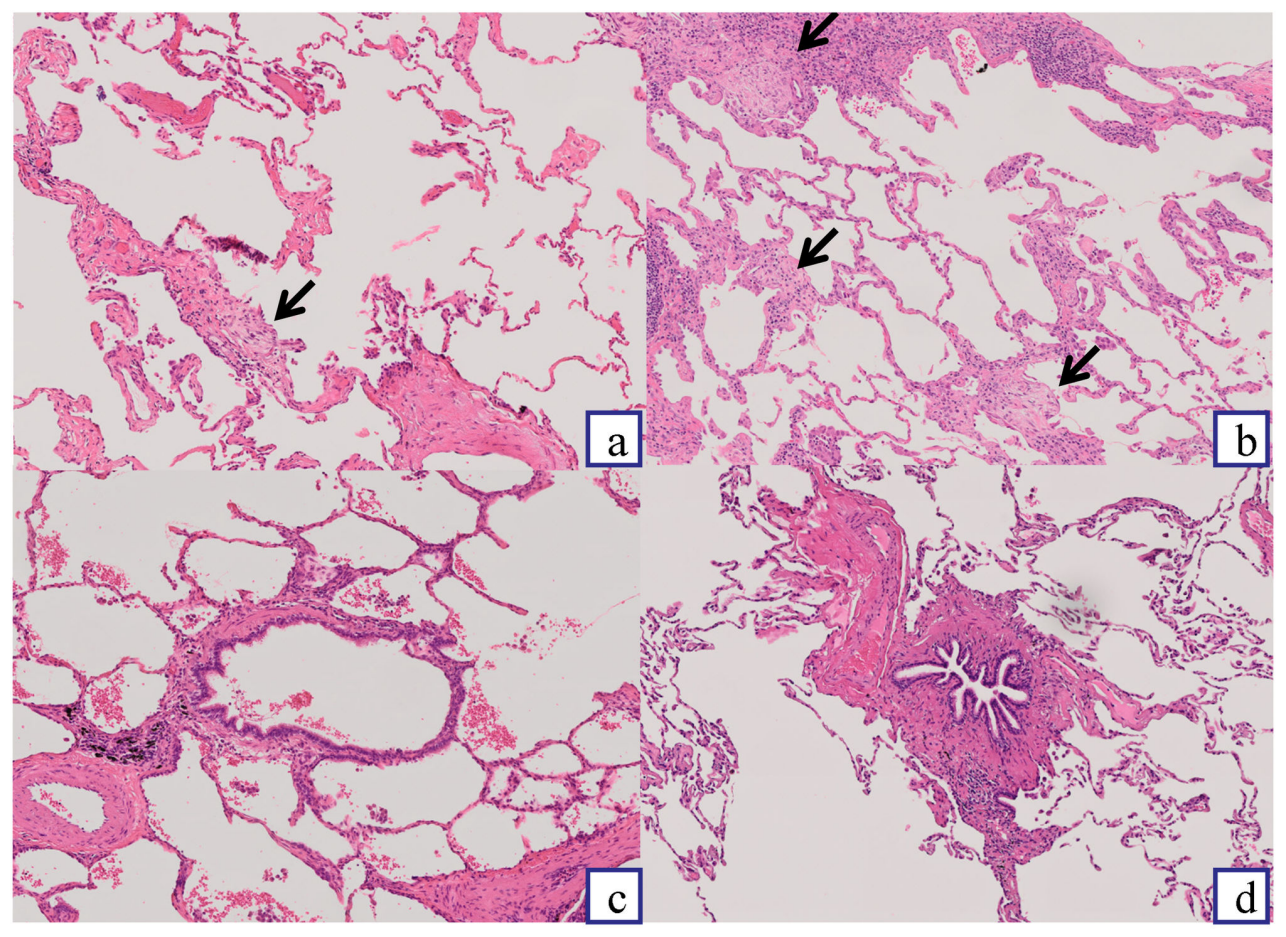
Examples of pathological scoring (hematoxylin–eosin, × 100). Typical images in each grade of fibroblastic foci (straight arrows) [(a): grade 1 and (b): grade 3] and bronchiolar fibrosis [(c): grade 1 and (d): grade 3] were listed.

Pathological findings are also summarized in Table 4. Various severities of cellular infiltration and fibrosis were often found not only in alveolar walls but also in small airways. The severity of microscopic honeycombing in the UIP patients was significantly higher than that in the NSIP patients (*P* < 0.01). The severities of fibroblastic foci in the UIP patients were relatively, but not significantly, higher than those in the NSIP patients. The typical examples of pathological features in each grade are shown in Figure 2.

### Outcome and Survival Analysis

The median follow–up period after surgical lung biopsy was 110 months (range: 2–159 months). One or more anti-inflammatory agents (corticosteroids, cyclosporin, azathioprine, or cyclophosphamide) were prescribed for 27 of the 33 patients. Only one patient was on corticosteroid therapy before lung biopsy. Long–term oxygen therapy was initiated in 11 of the 33 patients. There were no significant differences in the ratio of patients on medication or oxygen therapy between UIP and NSIP (data not shown).

**Figure 3:**
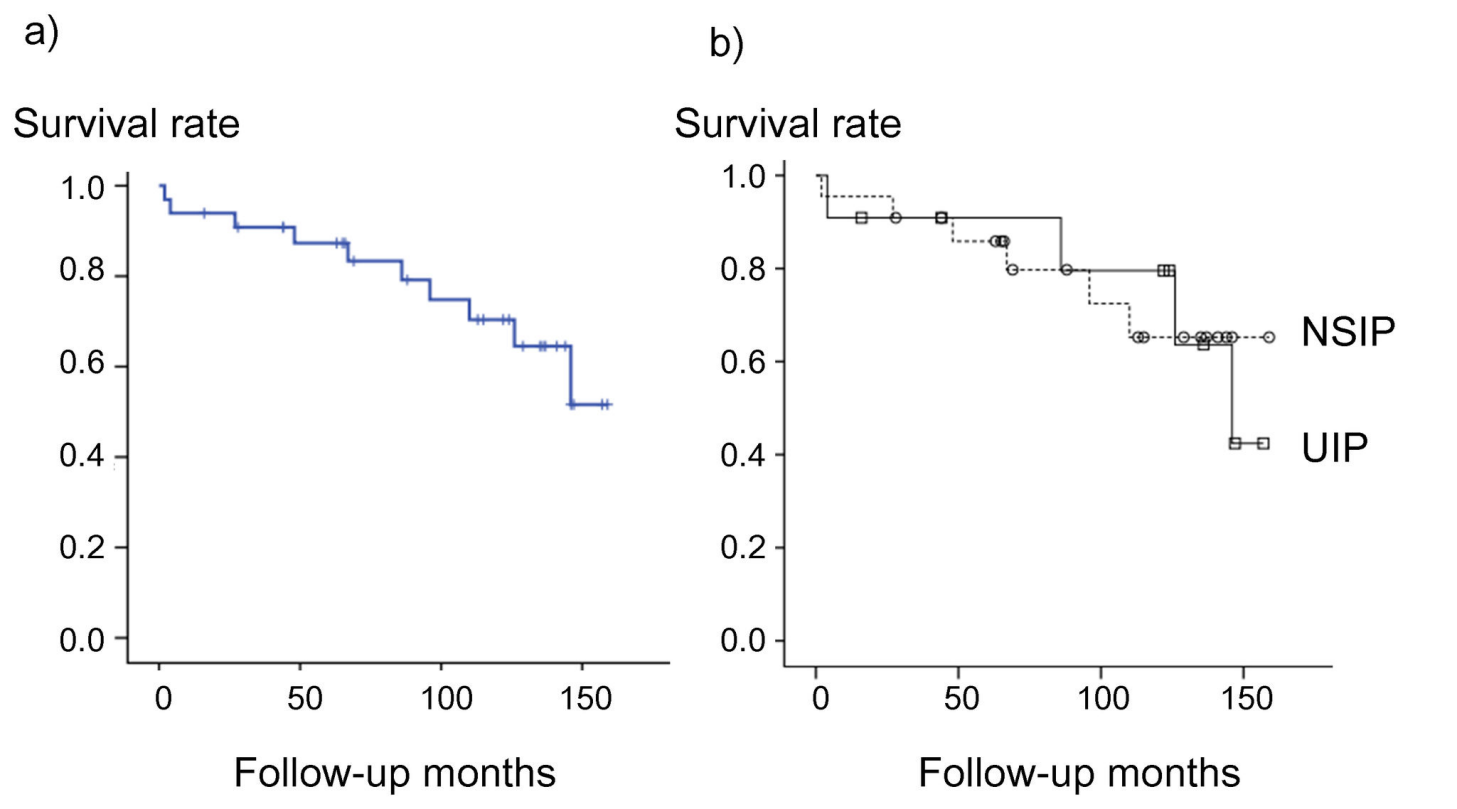
Kaplan–Meier survival curves for patients with interstitial lung disease associated with primary Sjögren’s syndrome. (a) Survival curve for the total patient population. Five-year survival rate was 87.3%. (b) Comparison of survival curves between the NSIP (n = 22) and UIP (n = 11) patients. Open circles or open squares show censored cases in NSIP group or UIP group, respectively. The prognosis between the two groups was not significantly different (*P* = 0.93 in log–rank test). UIP: usual interstitial pneumonia; NSIP: nonspecific interstitial pneumonia.

Ten of the 33 patients died, and five dropped out of follow–up. Causes of death were chronic respiratory failure (n = 5), acute exacerbation of ILD (n = 3), bacterial pneumonia (n = 1), and sepsis with unknown etiology (n = 1). No patients underwent lung transplantation. Survival at five years calculated by the Kaplan–Meier method was 87.3% in the total patient population (Figure 3a). Comparison of the survival curve between the NSIP and UIP patients is shown in Figure 3b. Five-year survival rates were 85.9% and 90.9% in the NSIP and UIP patients, respectively, and the survival difference was not statistically significant (*P* = 0.93 in log–rank test). The statistically significant difference was not proved in the prognosis between the two groups using Cox’s proportional hazards model with adjustment by baseline age and FVC % pred (NSIP to UIP, hazard ratio [HR]: 0.77, 95% confidence interval [CI]: 0.18–3.36, *P* = 0.73). In addition, we compared the prognosis between patients with pathological UIP in part or in whole (n = 16) and those without any fragment of UIP (n = 17) with adjustment by age and FVC % pred. There was no significant difference in their prognosis (HR: 0.78, 95% CI: 0.17–3.58, *P* = 0.75).

Clinical–radiologic–pathologic baseline variables were examined for influence on survival of pSS–ILD. The results of univariate analyses for the risk of death in the entire patient population are shown in Table 5. The following eight candidates of prognostic factors were found: age, PaCO_2_, FVC % pred, extent of ground glass attenuation on HRCT, extent of reticular abnormality on HRCT, pathological severity of interstitial fibrosis, fibroblastic foci, and bronchiolar fibrosis (all with *P* < 0.05). Sequentially, stepwise multivariate analysis was performed including those variables confirmed without close interactions (each absolute value of correlation coefficient < 0.7). PaCO_2_ (per 1 Torr increase, HR: 1.68, 95% CI: 1.24–2.28, *P* < 0.01), extent of reticular abnormality on HRCT (per 1-grade increment, HR: 4.17, 95% CI: 1.18–14.73, *P* = 0.03), and severity of fibroblastic foci (per 1-grade increment, HR: 9.26, 95% CI: 1.74–49.35, *P* < 0.01) were found to be independent and statistically significant prognostic factors.

**Table 5:** Univariate analysis for the risk of death in the 33 patients with interstitial lung disease associated with primary Sjögren’s syndrome.

**Variables**	**HR**	**95% CI**	***P***
**Clinical findings**			
Sex, female	0.77	0.22–2.76	0.69
Age*, per 1 year increase	1.24	1.05–1.47	0.01
Anti SS–A/Ro or SS–B/La antibody, positive	1.40	0.40–4.93	0.60
PaO_2_ (room air), per 1 Torr increase	0.95	0.86–1.05	0.30
PaCO_2_ (room air), per 1 Torr increase	1.18	1.02–1.36	0.03
FVC % pred, per 1% increase	0.97	0.94–1.00	0.03
**HRCT findings**			
Honeycombing, positive	1.13	0.29–4.47	0.86
Dilatation of pulmonary artery, positive	1.63	0.46–5.83	0.45
Ground glass attenuation, per 1-grade increment	2.42	1.14–5.12	0.02
Reticular abnormality, per 1-grade increment	3.04	1.42–6.50	< 0.01
Micronodules, per 1-grade increment	1.30	0.56–3.02	0.54
**Pathological findings, per 1-grade increment**			
Interstitial fibrosis	2.61	1.04–6.55	0.04
Fibroblastic foci	2.83	1.39–5.76	< 0.01
Microscopic honeycombing	1.73	0.94–3.17	0.08
Bronchiolar fibrosis	3.21	1.31–7.90	0.01
Vascular intimal or medial thickening	2.15	0.97–4.75	0.06

All *P* values were evaluated by Cox’s proportional hazards regression model. HR = hazard ratio; CI = confidence interval; PaO_2_ = arterial oxygen pressure; PaCO_2_ = arterial carbon dioxide pressure; FVC = forced vital capacity; pred = predicted; HRCT = high-resolution computed tomography.

* At the time of surgical lung biopsy.

## Discussion

In this study, we described the detailed characteristics of pathologically–proven pSS–ILD and diagnosed ILD pattern of each patient from multidisciplinary perspectives. NSIP was the most frequently observed ILD pattern, although UIP was not rare. Interestingly, prognostic difference between NSIP and UIP was not observed. Several candidates of prognostic factors in univariate analyses were found; of those, baseline PaCO_2_, extent of reticular abnormality on HRCT, and severity of fibroblastic foci were significantly related to prognosis in multivariate analysis.

The prognosis of pSS–ILD in our cohort was favorable. Pathological UIP pattern and NSIP pattern were coexistent in substantial amount of patients, and this was why we needed to conduct multidisciplinary diagnosis. UIP was not related to poorer prognosis compared with NSIP, even when the ILD having pathological UIP pattern in part or in whole were categorized as UIP. This result disagrees with a previous study for IIPs [18]. UIP in IIPs is well known as a determinant of poor prognosis, in contrast to the better prognosis of idiopathic NSIP [8]. The similar tendency was reported in ILD associated with rheumatoid arthritis [19]. On the other hand, previous studies for ILD associated with several collagen vascular diseases showed no significant difference in the prognosis between the UIP and NSIP patterns [20,21]. It is noteworthy that the UIP pattern is not always poorly prognostic in certain underlying diseases.

We identified three clinical–radiologic–pathologic prognostic factors in pSS–ILD: PaCO_2_, extent of reticular abnormality on HRCT, and severity of fibroblastic foci. Retention of PaCO_2_, or hypercapnia, can be caused by various hypoventilatory etiologies such as airway disorders, sleeping disorders, or neuromuscular disorders. In general, hypercapnia is a rare phenomenon for ILD alone, even in patients with severe pulmonary function [22]. Although the exact mechanism of hypercapnia in each patient was unknown, all our patients had no evidence of critical comorbidities other than pSS and the lung involvement. Therefore, the main cause was thought to be the effect of airway disorders coexisting with ILD. Shi et al [7] highlighted that small airway disorders were often coexistent with pSS–ILD, which is consistent with our results. Not only interstitial change but also airway disorders may have impact on the disease progression of pSS–ILD.

The extent of reticular abnormality on HRCT was identified as one of the prognostic factors in pSS–ILD. The similar relationships in other ILDs between the degree of reticulation or fibrosis on HRCT and the prognosis have been described in several past reports [12,14,23]. HRCT is now commonly used to diagnose and follow ILDs. It is valuable to evaluate the prognosis by a noninvasive method. On the other hand, the severity of fibroblastic foci, which can be evaluated by surgical lung biopsy, was also an independent risk factor for death. Fibroblastic foci are known as a manifestation of ongoing lung injury in UIP/IPF [24]. Previous studies showed that patients with idiopathic NSIP [25,26] or UIP associated with collagen vascular diseases [17,27] had fewer fibroblastic foci and improved survival compared with the UIP/IPF patients. We believe that the severity of fibroblastic foci is an universal prognostic factor in fibrotic and chronic ILDs regardless of the etiologies or ILD patterns.

There have been few studies focusing on the prognosis of pSS–ILD with pathological evidence of surgical lung biopsy. Ito et al [5] demonstrated in their study including 33 pSS patients that NSIP was the most common ILD pattern. They concluded that the prognostic factors were baseline low PaO_2_ and presence of microscopic honeycombing. The prognostic factors in their cohort and ours were discrepant. The main reason for this may be that their study did not exclude patients with diagnosis other than ILD, with shorter follow–up period than ours. For the analysis of pSS–ILD, our study could represent the true characteristics more accurately. Perambil et al [6] reported 18 patients with lung involvement by pSS. They emphasized that acute exacerbation could occur in pSS–ILD. Acute exacerbation of ILD associated with several collagen vascular diseases has been reported [28]. In our study, three patients (fibrotic NSIP, n = 2; UIP, n = 1) died from acute exacerbation. The risk factors of acute exacerbation in pSS–ILD were unknown and could not be evaluated in our study because of small number of the event. Further accumulation of data is required for the analysis of this severe event.

There are several limitations in this study. First, the retrospective nature of the study might lead to unexpected various bias and impacted the uniformity of the data available for evaluation. Second, small cohort size made it difficult to draw definite conclusions, although our cohort had relatively large number of patients compared with the previous studies for pathologically–proven pSS–ILD. Third, potential clinical factors in estimating the prognosis such as changes in pulmonary function and timing of therapy initiation were not evaluated in this study. Further prospective studies with a larger number of subjects are needed to resolve these problems.

In conclusion, UIP in pSS–ILD was not related to poorer prognosis than NSIP. The prognostic factors were identified to be baseline PaCO_2_, extent of reticular abnormality on HRCT, and severity of fibroblastic foci. Assessment of detailed clinical–radiologic–pathologic findings is more important than distinguishing UIP to evaluate prognosis in pSS–ILD. 

## References

[1] Gabriel SE, Michaud K (2009) Epidemiological studies in incidence, prevalence, mortality, and comorbidity of the rheumatic diseases. Arthritis Res Ther 11: 229. doi:10.1186/ar2669. PubMed: 19519924. 19519924 10.1186/ar2669 PMC2714099

[2] Palm O, Garen T, Berge Enger T, Jensen JL, Lund MB et al. (2013) Clinical pulmonary involvement in primary Sjögren’s syndrome: Prevalence, quality of life and mortality--a retrospective study based on registry data. Rheumatology. 52: 173–179. doi:10.1093/rheumatology/ket103. PubMed: 23192906. 23192906 10.1093/rheumatology/kes311

[3] American Thoracic Society/European Respiratory Society. International Multidisciplinary Consensus Classification of the Idiopathic Interstitial Pneumonias. This joint statement of the American Thoracic Society (ATS), and the European Respiratory Society (ERS) was adopted by the ATS board of directors, 6 2001 and by the ERS Executive Committee, June (2001 (2002) Am J Respir Crit Care Med. 165: 277–304

[4] Kokosi M, Riemer EC, Highland KB (2010) Pulmonary involvement in Sjögren syndrome. Clin Chest Med 31: 489–500. doi:10.1016/j.ccm.2010.05.007. PubMed: 20692541. 20692541 10.1016/j.ccm.2010.05.007

[5] Ito I, Nagai S, Kitaichi M, Nicholson AG, Johkoh T et al. (2005) Pulmonary manifestations of primary Sjögren’s syndrome: A clinical, radiologic, and pathologic study. Am J Respir Crit Care Med 171: 632–638. doi:10.1164/rccm.200403-417OC. PubMed: 15579729. 15579729 10.1164/rccm.200403-417OC

[6] Parambil JG, Myers JL, Lindell RM, Matteson EL, Ryu JH (2006) Interstitial lung disease in primary Sjögren syndrome. Chest. 130: 1489–1495. doi:10.1378/chest.130.5.1489. PubMed: 17099028. 17099028 10.1378/chest.130.5.1489

[7] Shi JH, Liu HR, Xu WB, Feng RE, Zhang ZH et al. (2009) Pulmonary manifestations of Sjögren’s syndrome. Respiration. 78: 377–386. doi:10.1159/000214841. PubMed: 19390161. 19390161 10.1159/000214841

[8] Raghu G, Collard HR, Egan JJ, Martinez FJ, Behr J et al. (2011) An official ATS/ERS/JRS/ALAT statement: Idiopathic pulmonary fibrosis: Evidence–based guidelines for diagnosis and management. Am J Respir Crit Care Med 183: 788–824. doi:10.1164/rccm.2009-040GL. PubMed: 21471066. 21471066 10.1164/rccm.2009-040GL PMC5450933

[9] Vitali C, Bombardieri S, Jonsson R, Moutsopoulos HM, Alexander EL et al. (2002) Classification criteria for Sjögren’s syndrome: A revised version of the European criteria proposed by the American-European consensus group. Ann Rheum Dis 61: 554–558. doi:10.1136/ard.61.6.554. PubMed: 12006334. 12006334 10.1136/ard.61.6.554 PMC1754137

[10] Hansell DM, Bankier AA, MacMahon H, McLoud TC, Müller NL et al. (2008) Fleischner society: Glossary of terms for thoracic imaging. Radiology. 246: 697–722. doi:10.1148/radiol.2462070712. PubMed: 18195376. 18195376 10.1148/radiol.2462070712

[11] Uffmann M, Kiener HP, Bankier AA, Baldt MM, Zontsich T et al. (2001) Lung manifestation in asymptomatic patients with primary Sjögren syndrome: Assessment with high resolution CT and pulmonary function tests. J Thorac Imaging. 16: 282–289. doi:10.1097/00005382-200110000-00009. PubMed: 11685093. 11685093 10.1097/00005382-200110000-00009

[12] Best AC, Meng J, Lynch AM, Bozic CM, Miller D et al. (2008) Idiopathic pulmonary fibrosis: Physiologic tests, quantitative CT indexes, and CT visual scores as predictors of mortality. Radiology. 246: 935–940. doi:10.1148/radiol.2463062200. PubMed: 18235106. 18235106 10.1148/radiol.2463062200

[13] Kazerooni EA, Martinez FJ, Flint A, Jamadar DA, Gross BH et al. (1997) Thin-section CT obtained at 10-mm increments versus limited three-level thin-section CT for idiopathic pulmonary fibrosis: Correlation with pathologic scoring. AJR 169: 977–983. doi:10.2214/ajr.169.4.9308447. PubMed: 9308447. 9308447 10.2214/ajr.169.4.9308447

[14] Lynch DA, Godwin JD, Safrin S, Starko KM, Hormel P et al. (2005) High–resolution computed tomography in idiopathic pulmonary fibrosis: diagnosis and prognosis. Am J Respir Crit Care Med 172: 488–493. doi:10.1164/rccm.200412-1756OC. PubMed: 15894598. 15894598 10.1164/rccm.200412-1756OC

[15] Cherniack RM, Colby TV, Flint A, Thurlbeck WM, Waldron J et al. (1991) Quantitative assessment of lung pathology in idiopathic pulmonary fibrosis. The BAL cooperative group steering committee. Am Rev Respir Dis 144: 892–900. doi:10.1164/ajrccm/144.4.892. PubMed: 1718192. 1718192 10.1164/ajrccm/144.4.892

[16] Song JW, Do KH, Kim MY, Jang SJ, Colby TV et al. (2009) Pathologic and radiologic differences between idiopathic and collagen vascular disease-related usual interstitial pneumonia. Chest. 136: 23–30. doi:10.1378/chest.08-2572. PubMed: 19255290. 19255290 10.1378/chest.08-2572

[17] Flaherty KR, Colby TV, Travis WD, Toews GB, Mumford J et al. (2003) Fibroblastic foci in usual interstitial pneumonia: idiopathic versus collagen vascular disease. Am J Respir Crit Care Med 167: 1410–1415. doi:10.1164/rccm.200204-373OC. PubMed: 12615630. 12615630 10.1164/rccm.200204-373OC

[18] Flaherty KR, Travis WD, Colby TV, Toews GB, Kazerooni EA et al. (2001) Histopathologic variability in usual and nonspecific interstitial pneumonias. Am J Respir Crit Care Med 164: 1722–1727. doi:10.1164/ajrccm.164.9.2103074. PubMed: 11719316. 11719316 10.1164/ajrccm.164.9.2103074

[19] Tsuchiya Y, Takayanagi N, Sugiura H, Miyahara Y, Tokunaga D et al. (2011) Lung diseases directly associated with rheumatoid arthritis and their relationship to outcome. Eur Respir J 37: 1411–1417. doi:10.1183/09031936.00019210. PubMed: 20884744. 20884744 10.1183/09031936.00019210

[20] Bouros D, Wells AU, Nicholson AG, Colby TV, Polychronopoulos V et al. (2002) Histopathologic subsets of fibrosing alveolitis in patients with systemic sclerosis and their relationship to outcome. Am J Respir Crit Care Med 165: 1581–1586. doi:10.1164/rccm.2106012. PubMed: 12070056. 12070056 10.1164/rccm.2106012

[21] Park JH, Kim DS, Park IN, Jang SJ, Kitaichi M et al. (2007) Prognosis of fibrotic interstitial pneumonia: Idiopathic versus collagen vascular disease-related subtypes. Am J Respir Crit Care Med 175: 705–711. doi:10.1164/rccm.200607-912OC. PubMed: 17218621. 17218621 10.1164/rccm.200607-912OC

[22] Javaheri S, Sicilian L (1992) Lung function, breathing pattern, and gas exchange in interstitial lung disease. Thorax. 47: 93–97. doi:10.1136/thx.47.2.93. PubMed: 1549829. 1549829 10.1136/thx.47.2.93 PMC463579

[23] Kocheril SV, Appleton BE, Somers EC, Kazerooni EA, Flaherty KR et al. (2005) Comparison of disease progression and mortality of connective tissue disease-related interstitial lung disease and idiopathic interstitial pneumonia. Arthritis Rheum 53: 549–557. doi:10.1002/art.21322. PubMed: 16082627. 16082627 10.1002/art.21322

[24] Katzenstein AL, Myers JL (1998) Idiopathic pulmonary fibrosis. Clinical relevance of pathologic classification. Am J Respir Crit Care Med 157: 1301–1315. doi:10.1164/ajrccm.157.4.9707039. PubMed: 9563754. 9563754 10.1164/ajrccm.157.4.9707039

[25] Nicholson AG, Fulford LG, Colby TV, du Bois RM, Hansell DM et al. (2002) The relationship between individual histologic features and disease progression in idiopathic pulmonary fibrosis. Am J Respir Crit Care Med 166: 173–177. doi:10.1164/rccm.2109039. PubMed: 12119229. 12119229 10.1164/rccm.2109039

[26] Harada T, Watanabe K, Nabeshima K, Hamasaki M, Iwasaki H (2013) Prognostic significance of fibroblastic foci in usual interstitial pneumonia and non-specific interstitial pneumonia. Respirology. 18: 278–283. doi:10.1111/j.1440-1843.2012.02272.x. PubMed: 23016880. 23016880 10.1111/j.1440-1843.2012.02272.x

[27] Enomoto N, Suda T, Kato M, Kaida Y, Nakamura Y et al. (2006) Quantitative analysis of fibroblastic foci in usual interstitial pneumonia. Chest. 130: 22–29. doi:10.1378/chest.130.1.22. PubMed: 16840378. 16840378 10.1378/chest.130.1.22

[28] Kondoh Y, Taniguchi H, Kitaichi M, Yokoi T, Johkoh T et al. (2006) Acute exacerbation of interstitial pneumonia following surgical lung biopsy. Respir Med 100: 1753–1759. doi:10.1016/j.rmed.2006.02.002. PubMed: 16584880. 16584880 10.1016/j.rmed.2006.02.002

